# Impact of Emphysema Heterogeneity on Pulmonary Function

**DOI:** 10.1371/journal.pone.0113320

**Published:** 2014-11-19

**Authors:** Jieyang Ju, Ruosha Li, Suicheng Gu, Joseph K. Leader, Xiaohua Wang, Yahong Chen, Bin Zheng, Shandong Wu, David Gur, Frank Sciurba, Jiantao Pu

**Affiliations:** 1 Department of Radiology, University of Pittsburgh, Pittsburgh, Pennsylvania, United States of America; 2 Department of Biostatistics, University of Pittsburgh, Pittsburgh, Pennsylvania, United States of America; 3 Peking University Third Affiliated Hospital, Beijing, People's Republic of China; 4 School of Electrical and Computer Engineering, University of Oklahoma, Norman, Oklahoma, United States of America; 5 Department of Medicine, University of Pittsburgh, Pittsburgh, Pennsylvania, United States of America; 6 Department of Bioengineering, University of Pittsburgh, Pittsburgh, Pennsylvania, United States of America; VU University Medical Center, Netherlands

## Abstract

**Objectives:**

To investigate the association between emphysema heterogeneity in spatial distribution, pulmonary function and disease severity.

**Methods and Materials:**

We ascertained a dataset of anonymized Computed Tomography (CT) examinations acquired on 565 participants in a COPD study. Subjects with chronic bronchitis (CB) and/or bronchodilator response were excluded resulting in 190 cases without COPD and 160 cases with COPD. Low attenuations areas (LAAs) (≤950 Hounsfield Unit (HU)) were identified and quantified at the level of individual lobes. Emphysema heterogeneity was defined in a manner that ranged in value from −100% to 100%. The association between emphysema heterogeneity and pulmonary function measures (e.g., FEV1% predicted, RV/TLC, and DLco% predicted) adjusted for age, sex, and smoking history (pack-years) was assessed using multiple linear regression analysis.

**Results:**

The majority (128/160) of the subjects with COPD had a heterogeneity greater than zero. After adjusting for age, gender, smoking history, and extent of emphysema, heterogeneity in depicted disease in upper lobe dominant cases was positively associated with pulmonary function measures, such as FEV1 Predicted (p<.001) and FEV1/FVC (p<.001), as well as disease severity (p<0.05). We found a negative association between HI% , RV/TLC (p<0.001), and DLco% (albeit not a statistically significant one, p = 0.06) in this group of patients.

**Conclusion:**

Subjects with more homogeneous distribution of emphysema and/or lower lung dominant emphysema tend to have worse pulmonary function.

## Introduction

As the most common phenotype of chronic obstructive pulmonary disease (COPD), emphysema is one of the leading cause of disability and death in the United States and worldwide [1]. This disease often goes undiagnosed for many years and irreversibly destroys lung parenchyma (alveoli), causes hyperinflation, and reduces lung elasticity [2]. In clinical practice, emphysema is typically diagnosed by pulmonary function tests (PFTs), medical history, and physical examination. However, traditional PFTs have a low sensitivity for diagnosing early stage COPD. It was reported that 30% of patients may have emphysema before exhibiting any detectable decline in pulmonary function [3]. In contrast, computed tomography (CT) is highly sensitive to depiction of the presence of emphysema. The extent of parenchyma destruction is often quantified as the fraction of lung voxels with Hounsfield Units (HU) value below a specific threshold (e.g., <−950 HU) [4]. Although pulmonary function and the amount of emphysema are significantly correlated, there is frequently a large variability in the computed fractional values of emphysema at a specific pulmonary function value and vice verse [5]. Hence, it is widely believed that spatial distribution and pattern of emphysema may be important for understanding the observed lack of concordance between pulmonary function and the amount of diseased lung.

Emphysema is widely classified into three pathological phenotypes: centrilobular, panlobular, and paraseptal [6]–[7]. It has been shown that panlobular emphysema was associated with *alpha 1*-antitrypsin deficiency, while centrilobuar emphysema was associated with tobacco exposure [8]–[9]. Recently, by classifying a population of 9,313 smokers into five subgroups in terms of emphysema patterns (i.e., mild centrilobular, moderate centrilobular, severe centrilobular, panlobular, and paraseptal emphysema), Castaldi et al. [10] showed that these phenotypes were strongly associated with a wide range of respiratory physiology and function measures. Other investigations assessed the spatial distribution of emphysema in different patient groups by dividing the lungs into: thirds [9], twelfths [8], and/or core and rind [11]. Different and at times conflicting findings have been reported [12]–[16]. However, all findings strongly and consistently suggest that the distribution patterns of emphysema have significant clinical implications and better understanding of the relationship between different patterns and lung function may aid in more precise patient stratification for optimal personalized management or treatment.

Anatomically the human lungs are comprised of five lobes that are serviced by their own of bronchovascular system and function somewhat independently from a mechanical, ventilation, and perfusion perspectives. Lobar independence may be what drives the different relations between pulmonary function and emphysema distribution and patterns. In particular, there are anatomical differences between the right and left lungs. For example, the right lung consists of three lobes, while the left lung is slightly smaller with only two lobes. Also, with respect to the trachea, the entering (branching) angle of the right bronchus is typically smaller than that of the left one because of the presence of the heart. Given the close relationship between structure and function, it is interesting to assess which lung tends to be more vulnerable to the disease. In this study, we quantified emphysema by lobes and investigated the possible impact of emphysema heterogeneity on pulmonary function in a well characterized cohort of participants in a chronic obstructive pulmonary disease (COPD) study.

## Methods and Materials

### A. Study population

**Figure 1:**
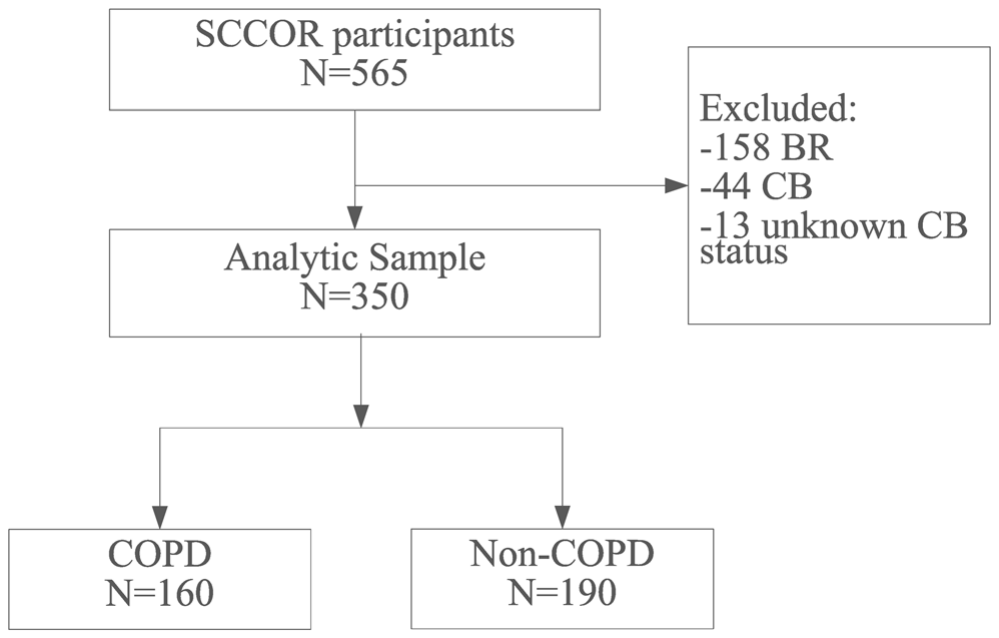
Flowchart illustrating how the final dataset analytic sample was obtained in which COPD by was defined by GOLD classification. (BR: subjects with bronchodilator response, and CB: subjects with chronic bronchitis).

We initially collected a dataset consisting of 565 CT exams from an NIH-sponsored Specialized Center for Clinically Oriented Research (SCCOR) in COPD at the University of Pittsburgh. The CT exams originate from the baseline data collection of the first 565 SCCOR participants who were recruited from those previously enrolled in the Pittsburgh Lung Screening Study (PLuSS) cohort [17]. The inclusion criteria for SCCOR were age >40 years and at least a 10 pack-year history of tobacco exposure. SCCOR participants underwent pre- and post-bronchodilator spirometry and plethysmography, measurement of lung diffusion capacity by single breath carbon monoxide (DLco), a chest CT examination and answered demographic as well as medical history questionnaires. The guidelines [18] published by American Thoracic Society were followed when performing these lung function tests. All data acquisition procedures and the CT scans were performed under a University of Pittsburgh Institutional Review Board-approved protocol (#0612016) with written informed consent obtained from all participants. The dataset included 346 subjects with COPD as defined by the Global Initiative for Obstructive Lung Disease (GOLD), and 219 subjects without COPD (Figure 1). Among the subjects with COPD, 137 had bronchodilator response (BR) and were excluded. An increase of 12% and 200 ml in the forced expiratory volume in one second (FEV1) was considered as a positive response to bronchodilator. We also excluded 49 patients who were either diagnosed with chronic bronchitis (CB) or had no related information on CB (Figure 1). CB was assessed based on series of questionnaires completed by the subjects and determined by whether there is a cough productive of sputum over three months' duration during two consecutive years and airflow obstruction. The demographics of the 160 subjects with COPD and the 190 subjects without COPD included in analysis are summarized in Table 1.

**Table 1:** Patient characteristics.

characteristics	All	COPD	non-COPD
	N = 350	N = 160	N = 190
**Sex: male** A	201(57%)	104(65%)	97(51%)
**Age (yrs)** B	64(61,69)	67(61,70)	63(60,67)
**Pack years, N = 287** B	50(35,70)	57(40,77)	45(32,63)
**FEV1%Predicted (%)** B	91(74,101)	71(48,85)	98(91,105)
**FEV1/FVC% (%)** B	72(62,77)	61(44,66)	77(75,81)
**RV/TLC (%)** B	38(34,43)	43(35,53)	36(33,40)
**DLco Predicted (%)** B	74(58,86)	61(42,75)	82(71,90)
**LAA%** B	1.2(0.5,4.4)	4.5(1.5,12.2)	0.5(0.2,1.3)
**HI%** B	17(4,36)	23(2,42)	14(4,27)
**GOLD classification** A			
None COPD	190(54%)	0(0%)	190(100%)
Gold I	55(16%)	55(34%)	0(0%)
Gold II	64(18%)	64(40%)	0(0%)
Gold III–IV	41(12%)	41(26%)	0(0%)

Categorical variables were summarized by frequency (%). Continuous variables were summarized by medians and interquartile ranges (IQR).

A Summarized by frequency (%);

B Summarized by median (IQR).

*Abbreviations*:

FVC – functional vital capacity.

FEV1% – forced expiratory volume in one second percent predicted.

RV –residual volume.

TLC –total lung capacity.

DLco% – diffusing lung capacity of carbon monoxide percent.

### B. Acquisition of thin-section CT examinations

CT examinations were acquired without the use of radiopaque contrast using a 64-detector CT scanner (LightSpeed VCT, GE Healthcare, Waukesha, WI, USA) while subjects held their breath at end inspiration. Scans were acquired using a helical technique at the following parameters: 32×0.625 mm detector configuration, 0.969 pitch, 120 kVp tube energy, 250 mA tube current, and 0.4-s gantry rotation (or 100 mAs). Images were reconstructed to encompass the entire lung field in a 512×512 pixel matrix using the GE “bone” kernel at 0.625-mm section thickness and 0.625-mm interval. Pixel dimensions ranged from 0.549 to 0.738 mm, depending on participant body size. The “bone” kernel was used because of its ability to visualize both parenchyma and airways [19]. The subjects were instructed in breathing to reach TLC prior to scanning; however, no real-time measures were employed to ensure breathing compliance such as spirometry-gated CT acquisition. The CT exams were reviewed to ensure compliance with the above mentioned chest CT protocol and were reviewed for artifacts that would contribute to poor image quality and/or quantification (e.g., subject motion and/or metal artifacts).

### C. Heterogeneity of emphysema distribution in different lobes

In-house developed software was used to segment the lungs and the lobes [20]–[22]. Similar to other investigations [23]–[24], low attenuation area (LAA) was defined as the volume of image voxels with HU values lower than −950 HU [25]. The percentage of low attenuation areas (%LAA) was computed as the sum of LAAs divided by the lung volume, and values were computed for the entire lung and each lobe. Small LAA clusters were removed to limit possible over-estimation of emphysema due to image noise and/or artifacts. Considering that CT images are reconstructed in a slice-by-slice manner and in-plane image pixel size ranges typically from approximately 0.65 mm to 0.90 mm, low attenuation areas with in-plane area less than 3 mm^2^ (approximately 4∼5 pixels in an image slice) were discarded and were not included in the %LAA computation. Considering that a median filter or other smoothing filters may alter pixel values, we chose not to use any smoothing filter. The clustering operation was performed after application of a threshold.

After the identification of regions associated with emphysema regions, an emphysema heterogeneity index (*HI*) between upper and lower lobes was computed (Eq. (1)), which results in values ranging from -100% to +100%. For the right lung, 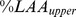 was computed as the sum of emphysema volume in the right upper and middle lobes. For the entire lung, 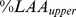 was computed as the combination of the emphysema in the left upper, right upper, and right middle lobes, while 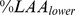 was the summed values for the left lower and right lower lobes. When emphysema is equally distributed among the lobes or its computed extent in the entire lung is less than 1%, *HI* has a zero value. 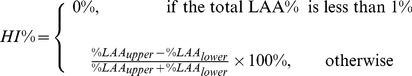
(1)


### D. Statistical analyses

We summarized the patient characteristics using the frequencies and proportions for categorical variables (e.g., sex and GOLD classification score) and median values and quartiles for continuous variables (e.g., age and FEV1% predicted). Considering that a large portion of patients may have emphysema but do not exhibit any detectable decline in pulmonary function [3], subgroups with COPD and without COPD were analyzed separately. We investigated the distribution of HI% and differences in HI% values between left lung and right lung using histograms. A one-sample chi-square test was used to examine whether the proportion of subjects with positive HI% was different from 50%. We used the sign test of location to examine whether the median difference in HI% values between the left and right lungs was different from zero.

The post-bronchodilator measurements used in the analyses include: (1) FEV1% predicted, (2) FEV1/FVC, (3) RV/TLC, and DLco%. We compared the distributions of these four pulmonary function measures between subjects with upper lung dominant emphysema (i.e., HI%>0%) and those with lower lung dominant emphysema (i.e., HI%≤0%) using the nonparametric Wilcoxon rank-sum test. In addition, we combined the GOLD classification score into two categories (I and II combined vs. III and IV combined) and compared the differences in distributions, if any, between the two groups.

To investigate the univariate association between HI% and lung function measures, we used piecewise linear regression method with a knot at 0, by defining HI%^+^ = max (0%, HI%) and HI%^−^ = min(HI%, 0%). The piecewise linear regression is a generalization of the conventional linear regression method, which corresponds to situations where the slopes of HI%^+^ and HI%^−^ are equivalent. Note that a piecewise linear regression model provides more flexibility in characterizing the relationship between HI% and outcomes of interest when compared to the conventional linear regression model. Next, we performed multiple linear regression modeling between HI% and the four lung function measures respectively, adjusting for age, sex, smoking history, and overall emphysema (LAA%). A multiple logistic regression model was adopted to model the odds of having higher GOLD classification results for GOLD III and IV combined, and the results were summarized in terms of odds ratios (OR). Statistical significance was defined by *p*<.05 (two sided). Analyses were performed using SAS (SAS Institute Inc, NC), version 9.3.

## Results

**Figure 2:**
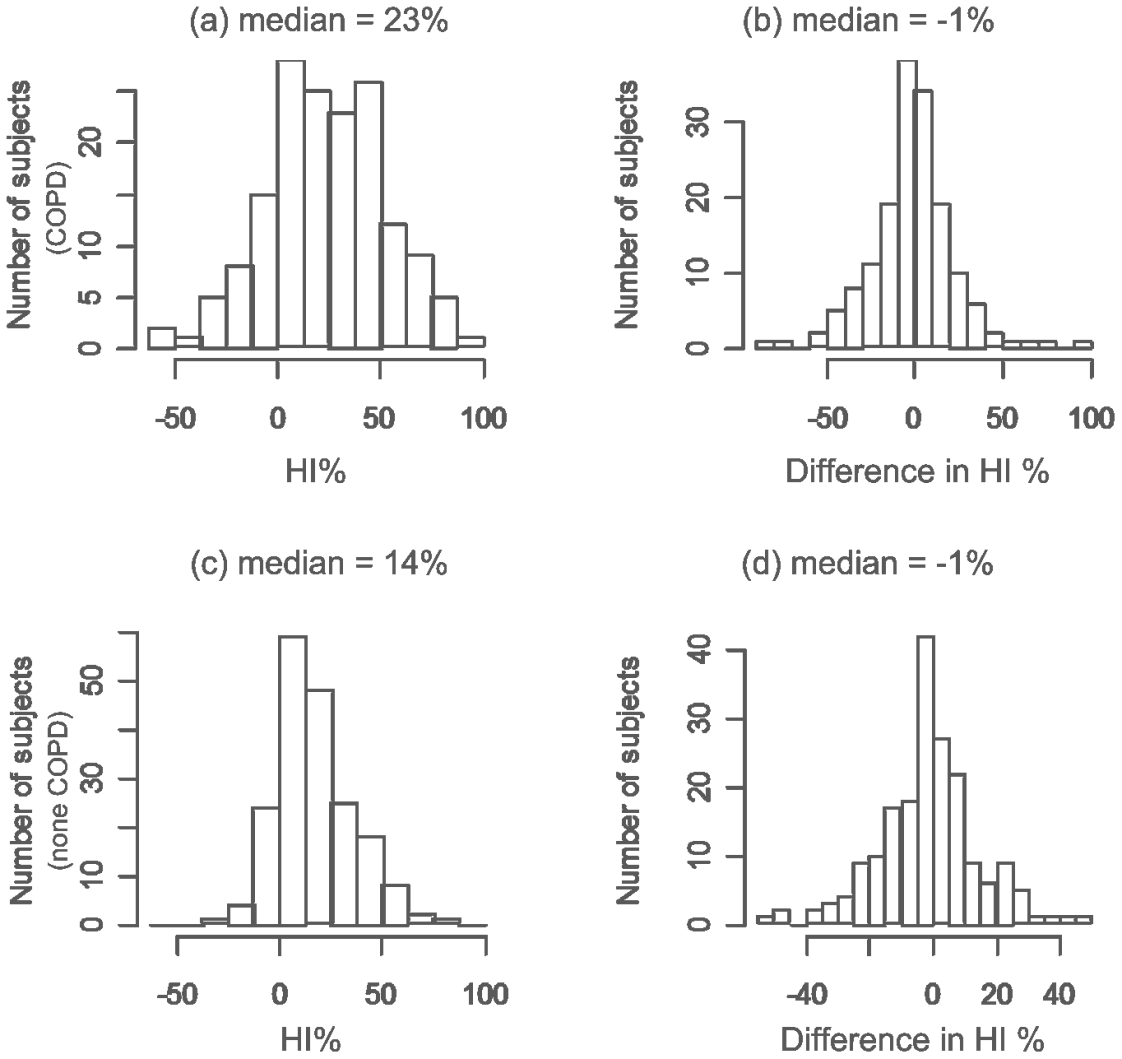
Distributions of computed HI% (left) and differences in computed HI% between the left and the right lungs (right). The top row represents the COPD patients (N = 160), and the bottom row represents the non-COPD patients (N = 190).

In the participants with COPD, median age was 67, 65% were male, median pack-years was 57, median FEV1% predicted was 71%, and median FEV1/FVC% was 61%. Forty-one (26%) of the COPD subjects had a GOLD classification scores greater than II (Table 1). In the participants without COPD, median age was 63, 51% were male, median pack-years was 45, median FEV1% predicted was 98%, and median FEV1/FVC% was 77%. Upper lobe dominant emphysema (positive HI%) was significantly more prevalent in both subjects with COPD (129/160 or 80.6%) and without COPD (161/190 or 84.7%) compared to lower lobe dominant emphysema (p<0.05 in both sets of subjects) (Figures 2A and 2C). The HI between the left and right lungs was not significantly different (p>0.05) in both sets of subjects (Figure 2B and 2D).

**Figure 3:**
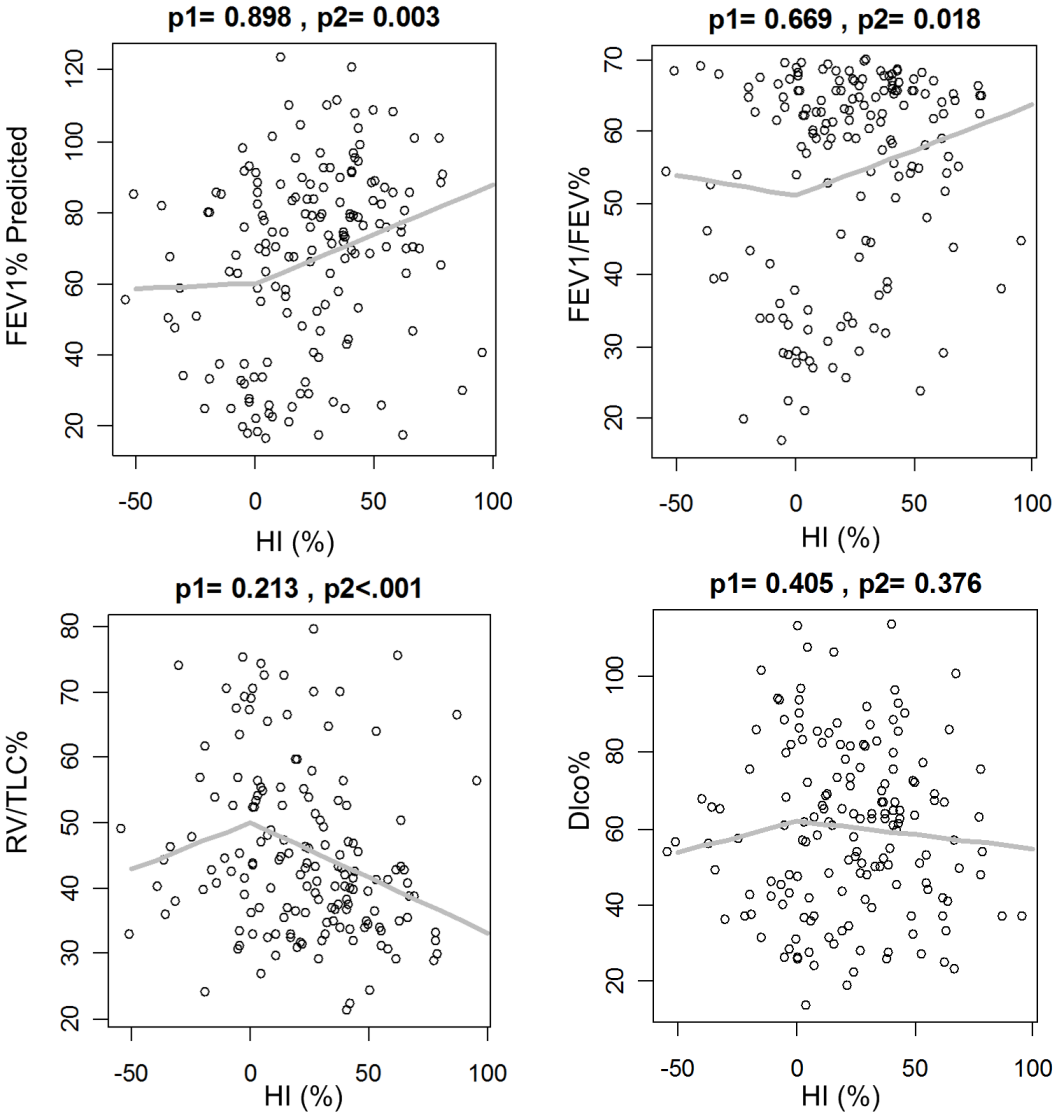
Scatter plots of pulmonary function measures and emphysema heterogeneity (HI%) for COPD patients. The grey line represents the estimated pulmonary function as a function of HI%. p1 denotes the p-value of HI%^−^ = min(0%, HI%) in the piecewise linear regression, and p2 corresponds to the p-value of HI%^+^ = max (0%, HI%).

Lung function in COPD subjects was significantly correlated with upper lobe dominant emphysema. Subjects with COPD and upper lung dominant emphysema (i.e., HI%>0) had significantly higher FEV1% predicted (p = 0.01) and, consequently, lower GOLD scores (p = 0.02) than COPD subjects with lower lung dominant emphysema (Table 2). In subjects without COPD and upper lobe dominant emphysema DLco% predicted was significantly lower than non-COPD subjects (Table 2). When HI was greater than zero (i.e., upper lobe dominant emphysema) it was significantly and directly correlated with FEV1 % predicted (p<0.001) and FEV1/FVC% (p = 0.002) and significantly and inversely correlated with RV/TLC% (p<0.001) among subjects with COPD (Figure 3). COPD subjects with lower lung dominant emphysema (i.e., HI%<0%) did not have a statistically significant correlation with lung function measures.

**Table 2:** Pulmonary function measures and COPD classification of subjects classified as having lower and upper lung dominant emphysema, stratified by emphysema heterogeneity and disease severity.

	COPD, N = 160			non-COPD, N = 190		
	HI< = 0%	HI>0%	p-value	HI< = 0%	HI>0%	p-value
	N = 31	N = 129		N = 29	N = 161	
**FEV1% Predicted (%)** B	55(32,80)	74(55,88)	0.01	99(91,108)	98(92,104)	0.8
**FEV1/FVC (%)** B	53(34,66)	62(51,66)	0.1	78(76,81)	77(75,81)	0.3
**RV/TLC (%)** B	45(39,57)	42(35,52)	0.1	35(34,40)	36(33,40)	0.9
**DLco Predicted (%)** B	56(42,76)	62(43,75)	0.7	89(83,92)	79(70,89)	0.01
**GOLD classification** A			0.02			
GOLD I–II	18(58%)	101(78%)				
GOLD III–IV	13(42%)	28(22%)				

A Summarized by frequency (%);

B Summarized by median (IQR).

Emphysema heterogeneity in COPD subjects significantly contributed to linear regression models of lung function that included overall emphysema (LAA%), HI, age, gender, and smoking history. Heterogeneity index significantly and positively contributed to the linear model of FEV1% predicted (p<.001) and FEV1/FVC% (p = 0.002) and significantly and negatively to the linear model of RV/TLC% (p<.001) among COPD subjects with upper lung dominant emphysema (Table 3). A one percent increase in HI was associated with 0.28% (95% CI 0.14–0.42%) increase in FEV1% predicted. For GOLD classification score, in a multiple logistic regression model of GOLD COPD classification including overall emphysema (LAA%), HI, age, gender, and smoking history, HI significantly and negatively contributed to GOLD classification with the probability of having higher GOLD classification scores (i.e. III or IV) among COPD subjects with upper lung dominant emphysema (Table 4). A one percent increase in HI resulted in decreasing the odds of having high GOLD classification score by a multiplicative factor of 0.953 (95% CI 0.91–0.99, p = 0.03). In a multiple linear regression model of subjects without COPD that included overall emphysema, HI, age, gender, and smoking history, HI% significantly and negatively contributed to RV/TLC%, but was not significantly associated with other lung function metrics (Table 5).

**Table 3:** Multiple linear regression results for different pulmonary function measures among subjects with COPD (n = 160).

	FEV1% Predicted Coef (CI)	FEV1/FVC% Coef (CI)	RV/TLC% Coef (CI)	DLCO% Coef (CI)
**LAA%**	−1.54 (−1.83,−1.26)	−1.09 (−1.22, −0.97)***	0.58 (0.43,0.74)***	−1.43 (−1.67, −1.18)***
**HI%^+^**	0.28 (0.14,0.42)***	0.09 (0.03,0.15)**	−0.15 (−0.23, −0.08)***	−0.11 (−0.23,0.01)
**HI%^−^**	0.02 (−0.29,0.33)	−0.03 (−0.17,0.11)	0.16 (−0.01,0.34)	0.19 (−0.08,0.46)
**Age (yrs)**	0.73 (0.17,1.29)*	0.06 (−0.18,0.31)	0.05 (−0.26,0.36)	0.18 (−0.3,0.66)
**Male**	9.53 (3.37,15.68)**	2.47 (−0.22,5.16)	−7.3 (−10.71, −3.88)***	6.46 (1.17,11.75)*
**Packyear**	−0.02 (−0.1,0.06)	0.00 (−0.04,0.03)	−0.02 (−0.07,0.02)	−0.01 (−0.08,0.06)

The cells represent the regression coefficients and estimated 95% confidence intervals (in parentheses).

*means 0.01≤p<0.05,

** means 0.001≤p<0.01, and *** means p<0.001.

**Table 4:** Multiple logistic regression model for GOLD classification score among subjects with COPD (n = 160).

	OR (CI)
**LAA%**	1.28 (1.16, 1.41)***
**HI%^+^**	0.95 (0.91, 0.99)*
**HI%^−^**	1.03 (0.94, 1.13)
**Age (yrs)**	0.88 (0.76, 1.02)
**Male**	0.25 (0.05, 1.29)
**packyear**	1.00 (0.98, 1.03)

The cells represent the estimated odds ratio (OR) and the corresponding confidence intervals.

**Table 5:** Multiple linear regression model for pulmonary function measures among subjects without COPD (n = 190).

	FEV1% Predicted	FEV1/FVC%	RV/TLC%	DLCO%
	Coef (CI)	Coef (CI)	Coef (CI)	Coef (CI)
**LAA%**	−0.82 (−2.17,0.53)	−0.78 (−1.23, −0.32)***	0.54 (−0.05,1.13)	−1.37 (−2.96,0.23)
**HI%^+^**	0.08 (−0.04,0.19)	−0.04 (−0.08,0)	−0.06 (−0.11, −0.01)*	−0.11 (−0.24,0.03)
**HI%^−^**	−0.4 (−0.88,0.09)	−0.03 (−0.2,0.13)	−0.01 (−0.22,0.21)	−0.6 (−1.18, −0.02)*
**Age (yrs)**	0.47 (0.04,0.9)*	−0.16 (−0.3, −0.01)*	0.43 (0.24,0.62)***	−0.27 (−0.78,0.24)
**Male**	2.27 (−1.57,6.1)	−0.3 (−1.59,0.99)	−5.38 (−7.06, −3.7)***	5.21 (0.66,9.76)*
**Packyear**	−0.04 (−0.11,0.03)	0 (−0.02,0.03)	0 (−0.03,0.03)	−0.09 (−0.17, −0.01)*

The cells represent the regression coefficients and estimated 95% confidence intervals (in parentheses).

## Discussion

The importance of emphysema distribution has been widely recognized. Accurate phenotypic description of emphysema may aid in predicting prognosis of emphysema and designing efficacy personalized therapy or management for preventing disease progression. In 1957, Leopold et al. [26] differentiated emphysema into two categories: centrilobular and panlobular. Presently, paraseptal emphysema is considered a third category of emphysema. This classification is based on the lobular locations of emphysema in the lungs. Castaldi et al. [10] found that emphysema phenotypes were strongly associated with respiratory physiology and function. Other investigators subdivided the lungs into varying number of zones and compared lung function across the zones [8]–[9], [11]. In contrast, we quantified emphysema by lobes and assessed the association between inter-lobar heterogeneity, COPD severity, and lung function. Unlike the traditional “gravitational” division of the lungs, this strategy takes the unique lung anatomy into account when characterizing the emphysema distribution. Three-hundred and fifty subjects were included in our analysis after exclusion of subjects with CB and positive bronchodilator response, which consisted of 160 subjects with and 190 subjects without COPD by GOLD classification. As compared to previous investigation [6]–[7], [27], this exclusion may enable more reliable assessment of the impact of emphysema on lung function. In our cohort, subjects with or without COPD and upper lobe dominant emphysema had significantly better pulmonary function compared with subjects with lower lobe dominant emphysema. However, smokers do not always have upper predominant emphysema. Also, subjects with or without COPD had similar levels of emphysema in the right versus left lungs despite the relatively large differences in anatomical structure.

Our findings agreed with Tanabe et al. [6] who reported that a more homogeneous distribution of emphysema was associated with an accelerated decline in FEV1 independently of baseline pulmonary function and overall emphysema severity. In their study, 131 male patients were involved. Similarly, Haraguchi et al. [27] reported similarly that uniformity of emphysema distribution correlated with severity of airway obstruction in pulmonary embolism (PE) patients, which is consistent with our finding in that subjects with lower lung dominant emphysema had significantly worse pulmonary function. According to this observation, the chance of a rapid decline in lung function is relatively lower in subjects with upper dominant and heterogeneous emphysema. In contrast, Gietema et al. [7] observed that an apical distribution was associated with more severe air-flow impairment when the emphysema was quantified at a threshold of −910 HU, but they found no significant impact when emphysema was quantified at a threshold of −950 HU.

As Tanabe et al. [6] explained, it is not easy to identify the exact factors behind the variety of emphysema distribution among smokers and explain why homogeneous distribution and lower lung dominant emphysema is critical for lung function. However, on the basis of previous investigations [13]–[14], where specific genes (e.g., MMP-9) were found to associate with upper lung dominant emphysema, we believe that specific genes could be associated with susceptibility to homogeneous emphysema. Also, given the close relationship between structure and function in the biological system, the morphology of specific structures (e.g., airway tree) that affect airflow could contribute to emphysema distribution as well, because airway morphology, which could be the result of specific genes, may alter the interaction between the harmful chemicals in the air and lung tissues. Therefore, in our opinion, a fully understanding of the variety of emphysema distribution may need multi-disciplinary investigative effort involving both imaging and genetics.

We note that there are limitations with this study. First, our study was performed at a single institution with a modest number of subjects and may or may not be generalizable to other cohorts or institutions. Although 350 subjects were included, the variety of emphysema extent and disease heterogeneity is still very limited. Non-smokers were not included. This data selection bias may somewhat affect the conclusion. To largely alleviate this issue, we proposed to exclude the asthmatic and CB patients to enable a relatively reliable analysis of the impact of emphysema heterogeneity on lung function. Second, the heterogeneity index may have oversimplified the complexity of heterogeneous distribution patterns of emphysema. Hence, more precise strategy for quantifying 3D distribution patterns of emphysema in the lung may be needed for improved phenotyping of emphysema. Third, we removed very small clusters of voxels below the threshold under the assumption they represent “image noises” or “artifacts.” However, further effort may be needed to assess whether the approach taken is optimal for this purpose. Fourth, the number of subject with lower lobe dominant emphysema was relatively small in both the COPD (n = 31) and non-COPD (n = 29) subjects, which may have hinder this phenotype from reaching statistical significance in some of the analyses. Finally, this retrospective cross-sectional study did not consider the impact of longitudinal changes in emphysema distribution patterns on pulmonary function or disease progression, but was outside the scope of this preliminary study.

## Conclusions

We investigated the association between inter-lobar heterogeneity of emphysema and pulmonary function measures. Our primary observation was that upper lone dominant emphysema was associated with significantly better lung function and significantly milder COPD compared to subjects with lower lobe dominant emphysema in both COPD and non-COPD subjects. We conclude that the chance of a rapid decline in lung function is relatively lower in subjects with upper dominant and heterogeneous emphysema.

## Supporting Information

Data S1 **Raw data in CSV format.**
(CSV)
